# The effect of simulation-based advanced cardiac life support training on nursing students’ self-efficacy, attitudes, and anxiety in Palestine: a quasi-experimental study

**DOI:** 10.1186/s12912-023-01588-z

**Published:** 2023-11-09

**Authors:** Maysa Fareed Kassabry

**Affiliations:** Nursing College, Arab American University, Arab American University- Palestine, P.O Box 240, 13 Zababdeh, Jenin, Palestine

**Keywords:** High-fidelity simulation, Advanced cardiac life support, Nursing student, Quasi-experimental

## Abstract

**Background:**

Cardiac Arrest (CA) is one of the leading causes of death, either inside or outside hospitals. Recently, the use of creative teaching strategies, such as simulation, has gained popularity in Cardio Pulmonary Resuscitation (CPR) instruction. This study aimed to assess the effect of High-Fidelity Simulation (HFS) training on nursing students’ self-efficacy, attitude, and anxiety in the context of Advanced Cardiac Life Support (ACLS).

**Methodology:**

The study design is quasi-experimental employing a pre-test and post-test approach during April and May 2023. A convenient sample of 60 undergraduate nursing students in a 4-year class from a nursing college at the Arab American University/ Palestine (AAUP) participated in this study. The data were analyzed using a paired sample t-test in SPSS program version 26. Three data collection tools were used pre- and post-intervention; the Resuscitation Self-Efficacy Scale (RSES), The Attitudinal instrument, and the State Anxiety Inventory (SAI).

**Results:**

The total number of nursing students was 60, out of them (56.7%) were female, while the mean age was (22.2) years. Improvements were seen in all four domains of self-efficacy following HFS training: recognition, debriefing, recording, responding and rescuing, and reporting. (t (59) = 26.80, *p* < 0.001, confidence interval [29.32, 34.05]). After receiving HFS training on ACLS, the post-intervention for the same group attitude scores significantly increased from 32.83 (SD = 15.35) to 54.58 (SD = 8.540) for emotion, from 6.72 (SD = 2.44) to 10.40 (SD = 1.40) for behavior, and from 7.03 (SD = 2.03) to 10.33 (SD = 1.42) for cognitive. The anxiety level decreased post-simulation from 3.53 (SD = 0.3) to 2.14 (SD = 0.65), which was found to be statistically significant (t(59) = 16.68, *p* < 0.001, 95% CI [1.22 to 1.55]). Female students (M = 73.18), students who observed a real resuscitation (M = 71.16), and who were satisfied with their nursing major (M = 72.17) had significantly higher self-efficacy scores post-simulation.

**Conclusion:**

The HFS can be recommended as an effective training strategy among nursing students. The ACLS training-based HFS was effective in improving the students’ self-efficacy and attitudes and decreasing their anxiety.

## Introduction

Cardiac Arrest (CA) is a critical condition where the heart’s activity suddenly stops, preventing it from pumping blood effectively and depriving the brain of oxygen [1]. A sudden emergency can occur anywhere, even in emergency rooms, inpatient departments, outpatient departments, and with patient families, visitors, and hospital staff at work. With over 500,000 deaths every year, CA is a leading cause of death worldwide [2]. Though there have been advancements in adult CA survival rates over the past two decades, the numbers remain alarming, with a mere 22% survival rate within hospitals and less than 10% outside hospital settings [3]. It is noteworthy that, despite standardization of fundamental cardiac resuscitation and post-arrest care, less than 10% of survivors are discharged from the hospital with positive neurological outcomes [4].

For the best care of patients experiencing CA, healthcare personnel are advised to complete Advanced Cardiac Life Support (ACLS) training. The ACLS encompasses a series of resuscitation, lifesaving drugs, electrical management, closed monitoring, and high-quality Cardio Pulmonary Resuscitation (CPR) [5]. The nurses’ proficiency in the life-saving emergency technique of cardiopulmonary resuscitation is what will allow them to react to a CA event swiftly and successfully [6]. Nurses are regarded as the most important members of the resuscitation team, and the effectiveness of CPR is greatly influenced by their expertise [7]. Many approaches exist for nurses to enhance resuscitation results. First, bedside nurses are frequently the first healthcare responders who can start resuscitation quickly, which has been associated with increased survival. Second, nurses lead the resuscitation team’s response before and after doctors arrive because they are members of fast response and resuscitation teams. Finally, nurses frequently play an important leadership role in initiatives to improve patient safety and hospital quality. Leaders in nursing can also encourage hospital-wide efforts and remove obstacles to care [8].

Nursing students should be taught the most current and appropriate information on resuscitation and be able to apply this theoretical knowledge in the real world. Recently, the use of innovative pedagogical approaches, such as simulation, has gained popularity in CPR instruction to enhance nursing students’ abilities and bolster their capacity for long-term knowledge retention. [9, 10]. Simulation as a teaching strategy encompasses many activities that simulate real-life clinical scenarios, aiming to demonstrate procedural skills, decision-making processes, and critical thinking abilities using role-playing, instructional media, models, and case studies [11]. Simulation-based education in nursing is a justifiable approach considering the challenges encountered in clinical settings, such as limited feedback, inadequate patient responses during exams, patient scarcity, and high student-to-patient ratios [12]. These creative methods of teaching have contributed to the improvement of students’ self-efficacy [13]. Participating in a simulation program increases nursing students’ confidence and their likelihood of performing CPR safely when confronted with a real patient. Studies have shown that simulation training has a favorable impact on student outcomes like knowledge acquisition, decision-making, self-efficacy, and self-confidence when used to prepare for clinical practice [14–16]. In pre-and post-test tests, Cant and Cooper discovered that simulation training statistically increased self-efficacy; also, in experimental designs, self-efficacy outperformed that of other teaching strategies [15]. Haddeland et al. found that the intervention group showed significantly larger progress when compared to the control group in a randomized controlled experiment comparing students’ knowledge and self-confidence levels before and after attending simulation training [16]. No significant impact on students’ self-efficacy or confidence was found in a systematic review and meta-analysis of the effectiveness of simulation training based on scenarios of life-threatening clinical conditions, but simulation training was found to be advantageous to other teaching techniques in terms of knowledge and performance improvement [17].

Simulation-based ACLS training is a viable and cost-effective method for transferring skills to trainees. It provides an optimal method for employers to evaluate how well their trainees are applying their skills and making decisions under simulated real-world conditions [18]. Utilizing simulation-based ACLS training will eventually enhance treatment quality and patient safety by increasing healthcare professionals’ commitment to evidence-based resuscitation techniques [19]. Additionally, by using a simulation approach, nurses may improve their performance, knowledge, and critical thinking while learning new professional skills without endangering the health of their patients [20]. The impact of simulation-based training on nurses’ abilities and knowledge was assessed in prior systematic review research, which showed that simulation training seems to be a useful tactic for enhancing nurses’ abilities [21]. Furthermore, a qualitative investigation carried out among Swedish midwifery students demonstrated that simulation facilitated the integration of theory and practice and offered a secure educational setting [22].

Simulation education is a powerful tool that not only improves self-efficacy among nursing students [23]; but also encompasses the cultivation of positive attitudes [24, 25] as well as the alleviation of anxiety [26]. These additional aspects are pivotal in shaping their proficiency in performing successful and precise cardiopulmonary resuscitation (CPR) [27], especially in regions like Palestine, which faces the challenges of being a conflict area with a high incidence of mass trauma-related casualties [28]. By promoting positive attitudes, students become more receptive to learning and willing to take on challenges [29]. With the challenging circumstances in Palestine, nursing students must possess a resilient attitude, recognizing the significance of their role in addressing the critical need for resuscitation care. Additionally, the reduction of anxiety helps students stay calm and focused during high-pressure situations, leading to improved performance and better patient outcomes [26].

Simulation-based training has the potential to provide researchers with valuable insights regarding the development of positive attitudes, reduction of anxiety, and enhancement of self-efficacy. These benefits ultimately contribute to the attainment of more effective training outcomes. By exploring the relationship between these concepts, nursing students can develop attributes such as successful and accurate resuscitations and ACLS, contributing significantly to saving lives and improving resuscitation care. These attributes empower nursing students in their future careers in Palestine as healthcare professionals.

High-fidelity simulation (HFS) is a popular training method in healthcare education [30], but limited research exists on its impact on nursing students’ self-efficacy, attitude, and anxiety during Advanced Cardiac Life Support (ACLS) training in Palestine. During ACLS simulations, students engage in intricate team dynamics, practicing essential skills such as leadership, communication, and teamwork within realistic ACLS scenarios. These simulations also involve unanticipated challenges introduced by instructors, promoting interaction, and adaptation [31]. This real-world environment not only introduces a new perspective to ACLS education but also serves as a fresh and novel method to enhance students’ self-efficacy and contributes valuable insights to the existing knowledge base on simulation-based ACLS training and its potential benefits. To the best of our knowledge, no prior studies have been conducted to evaluate the effect of High-Fidelity Simulation (HFS) training in Advanced Cardiac Life Support (ACLS) on nursing student`s self-efficacy, attitude, and anxiety. The absence of a similar study in the Palestinian context enhances the need for our study to enrich the literature and inform decision-makers about the current situation.

Therefore, this study aims to evaluate the effects of high-fidelity simulation (HFS) training on nursing students’ self-efficacy, attitude, and anxiety regarding advanced cardiac life support. Furthermore, the study aims to compare the outcomes between genders and to consider the impact of student nurses’ observation of practical CPR as well as their satisfaction with their major.

## Methodology

### Design

This study employed a quasi-experimental design using a pre-test and post-test approach with a single group. In the pre-test stage, all nursing students were asked to complete the self-efficacy, attitude, and anxiety tools. To assess the effect of HFS-based training, all students completed the same pre-test and post-test tools. The training was conducted over two consecutive days. The pre-test was administered on the first day of pre-intervention, while the post-test was held on the second day, after the intervention.

### Sample and participants

A convenient sample of undergraduate nursing students in a 4-year class from a nursing college at the Arab American University/ Palestine (AAUP) participated in this study during April and May 2023. To ensure meaningful analysis and the ability to generalize findings, a larger sample size was deemed necessary for this study. Using a single group design and a paired sample t-test, it was estimated that 52 participants would be required based on a medium effect size (d) of 0.40, [32, 33], 80% power, and a significance level of 0.05. This estimation was calculated using G*Power software version 3.1.9.7. To account for potential dropouts, an additional 8 participants were included, resulting in a final participant count of 60 for the study. The effect size was calculated using a pooled effect size for several approaches to learning in a comprehensive review and meta-analysis of instructional design aspects in simulation-based education [34].

All nursing students who completed their basic theoretical and practical training in adult health nursing and recently in advanced adult nursing science and had the willingness to participate in this study were included. Students suffering from chronic diseases, orthopedic disorders, upgraded students, or whoever participated in ACLS high-fidelity simulation were excluded from the study.

### The advanced cardiac life support (ACLS) training and high-fidelity simulation

The (ACLS) training took place in the American Heart Association Center (AHA) at the AAU and according to AHA guidelines [35] The training lasted for two consecutive days, 6 h per day. The ACLS training program consisted of course material that covered the core concepts and skills needed to manage emergency cardiac situations. The ACLS training program covered a range of topics, including recognition and management of cardiac arrest, airway management, use of advanced cardiovascular life support drugs, electrical therapy, and post-cardiac arrest care. The course material included instructional videos, lectures, case scenarios, and interactive simulations. The ACLS training was conducted with different formats, starting with a mandatory online pre-assessment exam as an essential component of the ACLS course followed by classroom-based training, watching tutorial videos, in-person training typically involves hands-on practice of skills using simulation equipment/ manikins and final pencil post-test exam. The simulation session took place in the simulation lab at the AAUP. It was based on a high-fidelity simulator and included many features such as a monitor screen showing all vital signs, palpable pulse, Electrocardiogram (ECG) monitoring, chest expansion, and simulation sound. The sixty students who participated in the simulation experience were divided into 10 groups, six students in each group. The ACLS and simulation were implemented by the principal investigator (MK) who is an ACLS-certified expert. At the end of the two-day sessions, all students shared teamwork emergency mega-code scenarios for 25 min per each using a performance checklist developed by AHA.

### Data collection tools

The researcher utilized four instruments to assess the effect of simulation on the nursing students` behavior when confronted with ACLS scenarios.

#### Demographic and clinical-related experiences

The first instrument focused on socio-demographic factors such as sex, age, grade point average (GPA), and clinical-related experiences such as satisfaction with their major, and experience of observing real CPR training, including the number of times they observed it.

#### Resuscitation self-efficacy scale (RSES)

The Resuscitation Self-Efficacy Scale (RSES) is used as a self-appraisal tool to assess the self-efficacy of nurses who have completed the CPR program [36]. The RSES encompasses 17 items measuring four domains; recognition (4 questions), debriefing and recording (4 questions), responding and rescuing (5 questions), and reporting (4 questions). A 5-point Likert-style scale was used to assess nursing students’ responses. The Scores ranged from 1 (least confident) to 5 (extremely confident) with higher ratings indicating stronger perceived self-efficacy, with total scores ranging from 17 to 70. The RSES was created as a valid instrument for measuring the self-efficacy of nurses in resuscitation situations [36, 37]. The internal consistency of the RSES showed a high value with Cronbach’s coefficient of 0.91 [36], while in this study Cronbach’s coefficient was 0.8.

#### Attitude

Attitude was the third instrument developed by Cho [38] referring to the guidelines set forth by both the KACPR (Korean Association of Cardiopulmonary Resuscitation) and AHA. A pilot study was conducted before the initiation of the actual study, which involved 10 nursing students (excluded from the study); the results of this pilot study confirmed that the research design and procedures were feasible. The attitude instrument was originally developed in Korean, but an English version was adopted from prior research [39]. Two experts confirmed the comprehensiveness and accuracy of the tool’s translation into English and subsequently back into Korean. The translated tool underwent face validity and content validity assessments with five experts. Face validity ensured its relevance and clarity with the construct being measured, while content validity ensured that the translated tool correctly captured the necessary content related to the construct being measured. Arrangements were made based on the recommendations of experts. The attitudinal instrument includes three types; emotional, behavioral, and cognitive attitude The Cronbach alpha values in the pilot study for emotional, behavioral, and cognitive attitudes were 0.93, 0.75, and 0.69, respectively.

The emotional attitude reflects an individual’s feelings towards performing basic CPR in the event of cardiac arrest (CA) and consists of 10 questions scored on a 7-point Likert scale (from 1 to 7), with a total score ranging from 7 to 70. Behavioral attitude measures the potential practice of performing CPR in the event of cardiac arrest (CA) and consists of 3 questions scored on a 4-point Likert scale (from 1 to 4), with a total score range from 3 to 12. The cognitive attitude measures individuals’ perceived capacity to perform CPR in the event of CA and includes 3 questions rated on a 4-point Likert scale (from 1 to 4), with a total score ranging from 3 to 12. The overall score goes from 0 to 94, with a higher number reflecting a more positive attitude. According to Cho’s study [38], the emotional, behavioral, and cognitive attitudes had a Cronbach’s alpha of 0.69, 0.77, and 0.63 respectively. In this study, the emotional, behavioral, and cognitive attitudes had Cronbach’s alphas of (0.96), (0.72), and (0.62) respectively. Knowing that values over 0.6 demonstrate valid, reliable, and satisfactory internal consistency [40, 41]. Also, in this study, it is noteworthy that nursing students received their initial ACLS instruction. The Cronbach’s alpha for cognitive attitudes was found to be lower in comparison to emotional and behavioral attitudes. This suggests a decline in ACLS comprehension and approach among students as the complexity of training increases. The addition of challenging scenarios and unanticipated events to their training made it more difficult for them to maintain a consistent cognitive attitude toward ACLS procedures. However, retraining these students may increase their Cronbach’s alpha value, resulting in a greater understanding of theory and application.

#### State-trait anxiety inventory- state (STAI-S)

The fourth instrument was the state anxiety subscale of the State-Trait Anxiety Inventory (SAI) [42]. The State-Trait Anxiety Inventory (STAI) is a psychological instrument that assesses both state and trait anxiety. It consists of 40 self-report items. State anxiety refers to a transient state of anxiety (20 questions), whereas trait anxiety (20 questions) refers to a more general and persistent character of anxiety. In the current study, the State Anxiety Inventory, a commonly utilized tool for assessing state anxiety, was employed [43]. The SAI (State-Trait Anxiety Inventory) is one of the most effective tools for evaluating anxiety, regardless of gender or race. Since 1983, SAI has been utilized in more than 200 investigations each year [44]. The researcher used the 20-item state anxiety scale Form-Y (SAI) of the STAI to assess anxiety levels in terms of how the nursing student feels “right now” in the before and after simulation. On a 4-point scale with options of “1 = not at all, 2 = somewhat, 3 = moderately, or 4 = very much”, students were asked to indicate how much their anxious sensations are affecting them. The validity and reliability tests of SAI were conducted by Oner and Le Compte (1983). The SAI for state anxiety showed high reliability with Cronbach’s coefficients for college-aged males at 0.91 and females at 0.93. While the Cronbach alpha in the present study was 0.91. The weighted scores ranged from 20 to 80, and the higher the weighted value, the more psychological stress [44].

### Data analysis

The SPSS program version 26 was used to analyze the entered data. Descriptive statistic was used to show the mean, Standard Deviation (SD), frequencies, and percentages. Inferential statistics were used as paired t-tests to compare the difference in self-efficacy, attitude, and anxiety before and after the simulation, after ensuring that the underlying statistical assumptions were met.

### Ethical considerations

Ethical approval was obtained from the IRB of the American Arab University, Palestine IRB (2023/A/48/N). All procedures were performed following the Declaration of Helsinki guidelines. Before beginning the research, all potential student participants were provided with a concise informed consent form that outlined the purpose of the study, the procedures involved, and the voluntary nature of their participation. The consent form also states explicitly that students have the right to decline or withdraw their participation at any time during the study without confronting negative consequences or having their academic scores affected.

## Results

The total number of nursing students was 60 with a (100%) response rate for those who participated in the pre-test, simulation-based training, and post-test.

### Demographic and clinical-related experiences

About half of the students were female (56.7%) and the mean age of study students was 22.2 years (SD: 0.54). Most of the nursing students were satisfied with their major (68.3%). The majority of students (81.7%) observed a real CPR, out of them (55.1%) observed the resuscitation for one time and (36.7%) observed the resuscitation two times. Table 1 shows the description of students’ demographics and clinical-related experience.

**Table 1:** Participants’ demographics and clinical-related experience *(N = 60)*

Variable	*n* (%)
**Age, mean (SD)**	22.24 (0.54)
**Gender**	
Male	26 (43.3)
Female	34 (56.7)
**Satisfaction with Major**	
Satisfied	41 (68.3)
Dissatisfied	19 (31.7)
**Have you ever observed a real resuscitation?**	
Yes	49 (81.7)
No	11 (18.3)
**If yes, what number of times have you observed**,	
One time	27 (55.1)
Two times	18 (36.7)
Three times or more	4 (8.1)

SD standard deviation

### Changes in nursing students’ self-efficacy, attitude, and anxiety levels

The study participants were asked to provide self-reported data on their perceived efficacy, attitude, and anxiety levels. To evaluate the impact of ACLS simulation education on these factors, a paired t-test was conducted. The results of the analysis, presented in Table 2, illustrate the changes observed in students’ self-efficacy and attitude after the implementation of ACLS simulation education. Despite the participants demonstrating high levels of self-efficacy across various dimensions during the pretest phase, notable improvements were observed in all dimensions of self-efficacy from the pretest to the posttest. Similarly, the overall RSES (Resuscitation Self-Efficacy Scale) score exhibited a noteworthy and statistically significant improvement in participants’ self-efficacy following the HFS implementation (t (59) = 26.80, *p* < 0.001, 95% CI [29.32–34.05]).

**Table 2:** Difference between the participants’ pre-test and post-test scores of their self-efficacy, attitude and anxiety (*N* = Table 2 Difference between the nursing students’ pre-test and post-test scores of their self-efficacy, attitude, and anxiety (*N* = 60)

Self-efficacy Dimensions	*M*	*SD*	*t*	*SE*	*p*	95% Confidence Interval of the Difference	
						**Lower**	**Upper**
Recognition (4 items)							
Pretest	8.73	2.239	20.30	0.38095	< 0.000**	6.97	8.50
Posttest	16.47	2.087					
Debriefing and recording (4 items)							
Pretest	8.50	1.935	24.05	0.33821	< 0.000**	7.46	8.81
Posttest	16.63	1.895					
Responding and rescuing (5 items)							
Pretest	12.33	3.448	15.85	0.54460	< 0.000**	7.54	9.72
Posttest	20.97	2.050					
Reporting (4 items)							
Pretest	9.05	2.770	13.86	0.518	< 0.000**	6.15	8.22
Posttest	16.23	2.173					
Total RSES score^a^							
Pretest	38.62	6.742	26.80	1.182	< 0.000**	29.32	34.05
Posttest	70.30	5.970					
Attitude Dimension							
Emotional attitude^b^ (10 items)							
Pretest	32.83	15.35	9.63	2.258	< 0.000**	17.23	26.27
Posttest	54.58	8.540					
Behavioral attitudes^c^ (3 items)							
Pretest	6.72	2.44	9.58	0.384	< 0.000**	2.91	4.45
Posttest	10.40	1.40					
Cognitive attitudes^d^ (3 items)							
Pretest	7.03	2.03	11.07	0.298	< 0.000**	2.70	3.90
Posttest	10.33	1.42					
Anxiety dimension^e^ (20 items)							
Pretest	3.53	0.30	16.68	0.083	< 0.000**	1.22	1.55
Posttest	2.14	0.65					

CI = confidence interval; RSES = Resuscitation Self-Efficacy Scale ^a^Range for the total score, 16–80. ^b^Range for the total score, 10–70.^c^Range for the total score, 3–12. ^d^Range for the total score, 3–12. ^e^Range, 20–80. ^*^Correlation is significant at the 0.05 level (2-tailed),^**^Correlation is significant at the 0.01 level (2-tailed)

Moreover, the paired t-test analysis demonstrated a significant improvement in all attitude domains during the post-test assessment. Specifically, the emotional attitude domain displayed a notably higher mean score of 54.58 after the intervention compared to the pre-intervention score of 32.83. This substantial mean difference of 21.75 was found to be statistically significant (t(59) = 9.63, *p* < 0.001, 95% CI [17.23–26.27]). Also, after the intervention, participants scored a much higher mean of 10.40 on the behavioral attitude dimension, compared to 6.72 before. Significant statistical significance was discovered for the large mean difference of 3.68 (t(59) = 9.58, *p* < 0.001, 95% CI [2.91–4.45]). Similarly, in the cognitive attitude dimension, participants displayed a substantial increase in their mean score after the intervention, with a higher score of 10.33 compared to 7.03 before. This significant improvement was supported by statistical analysis, revealing a large mean difference of 3.3, (t(59) = 11.07, *p* < 0.001, 95% CI [2.70–3.90]). On the contrary, the paired t-test analysis revealed a significant decrease in anxiety levels during the post-test assessment (M = 3.53), in comparison to the pre-intervention anxiety levels (M = 2.14) in the HFS intervention. The mean difference of 1.39 was found to be highly significant (t(59) = 16.68, *p* < 0.001, 95% CI [1.22–1.55]).

In Table 3, the association between gender and post-test RSES total score revealed significantly higher self-efficacy scores among females (M = 73.18) than their male counterparts (M = 66.54). The mean difference of 6.64 is statistically significant (t(58) = 5.09, *p* < 0.001, 95% CI [9.25–4.03]). Moreover, the analysis shows that the student nurses who have ever observed a real CPR had a significantly higher mean score of self-efficacy (M = 71.16) compared with those who hadn’t such an observation (M = 66.45). The mean difference of 4.71 is statistically significant (t(58) = 2.46, *p* < 0.05, 95% CI [0.88–8.53]). The results also indicate that there is a significant association between satisfaction with major and the post-test RSES total score. Those who reported being satisfied (M = 72.17) displayed significantly higher self-efficacy scores compared to those who reported being unsatisfied (M = 66.26), with a mean difference of 5.91. This difference was found to be statistically significant, with t(58) = 3.99, *p* < 0.001, 95% CI (2.94 to 8.87).

**Table 3:** T-test analysis of the Posttest self-efficacy with gender, CPR observation, and satisfaction with major

Variable		Mean	SD	t-test	P-value
Gender	**Male**	66.54	3.49	5.09	< 0.001**
	**Female**	73.18	5.90		
CPR Observation	**Yes**	71.16	5.93	2.46	< 0.05*
	**No**	66.45	4.63		
Satisfaction with major	**Satisfied**	72.17	5.72	3.99	0.001**
	**Dissatisfied**	66.26	4.34		

^*^Correlation is significant at the 0.05 level (2-tailed) ^**^Correlation is significant at the 0.01 level (2-tailed)

## Discussion

To the best of the author’s knowledge, this is the first study aimed at assessing the effect of HFS training about ACLS on nursing students’ self-efficacy, attitude, and anxiety levels. This study found that the mean score of the RSES has been increased after the intervention. This implies that the HFS was significantly effective in boosting the self-efficacy of the nursing students. Such a result was supported by previous studies with either similar objectives and similar or different nursing topics [26, 45–49]. A quasi-experimental study conducted among 62 nurses to examine the effect of HFS education on self-efficacy in ACLS, found that the total RSES score was significantly improved post-simulation [47]. A randomized controlled trial to compare the effectiveness of HFS and traditional teaching methods among nursing students on cardiac auscultation concluded that the use of HFS was more effective than the traditional method in improving the students’ skill levels for cardiac auscultation [26]. A recently published quasi-experimental study evaluating the impact of traditional instruction versus simulation on nursing students’ capacity for self-efficacy showed significantly higher post-test self-efficacy scores [45]. Nursing students who participate in simulation programs have increased confidence and proficiency in performing CPR on actual patients [50]. Higher self-efficacy in nurses demonstrates a greater capacity for behavior management and role competence [51]. However, in this study, the finding could be attributed to the high self-confidence gained by students who were able to clinically practice CPR activities similar to the real situation. This study also revealed a significantly higher self-efficacy among students who had ever observed a real CPR (Mean = 71.16, SD = 5.93). Contrary to the researcher’s expectation, the analysis showed that self-efficacy was higher among female students (M = 73.18, SD = 5.90, *P* < 0.001) than among their counterparts. A newly published experimental study aimed at comparing nursing student self-efficacy post-simulation–clinical training found there was no significant difference between male and female nurses [46]. However, one possible explanation for the study`s finding is that female students may have more theoretical knowledge which made their capabilities and self-confidence higher than male students [52]. Another important finding pointed out that student nurses who were satisfied with their major had significantly higher post-test total RSES scores (M = 72.17, SD = 5.72). Such a finding reflects how much the satisfaction of a nursing major can motivate students to have better abilities and skills in clinical practice. This explanation is supported by a study that concluded a positive association between student nurses’ satisfaction in nursing program/clinical learning environments and their self-efficacy [53].

In this study, the analysis showed a post-test significant improvement in emotional, behavioral, and cognitive attitudes. This implies that HFS training can be effective in positively improving the students’ attitudes by influencing their emotions and capabilities toward implementing CPR. Such a finding was highly expected and can be explained by the view that hands-on training can refine clinical skills and thus ameliorate students’ emotional and behavioral attitudes. The current study result is also congruent with a randomized controlled trial that assessed the effect of blended CPR training on nursing student attitudes and revealed that the interventional group had significantly higher post-test behavioral and cognitive attitudes [39]. The impact of high-fidelity simulation training on knowledge, practice, and attitude was also assessed for 100 third-year pediatric nursing students at Benha University regarding cardiopulmonary resuscitation by Mohamed et al. (2017). The results demonstrated a noteworthy improvement in the total knowledge, attitude, and practice scores of the students after the training, indicating that high-fidelity simulation training is an effective method for enhancing the knowledge, practice, and attitude of nursing students [54]. Therefore, these findings provide additional evidence supporting the integration of high-fidelity simulation training as a valuable approach to nursing education. These outcomes also align with the conclusions drawn by Gyung Park’s (2015) study which found that basic CPR training significantly improved participants’ attitudes and self-efficacy scores, positively impacting their mindset and confidence in performing CPR. The training improved both attitudes and competency, increasing their chances of success in performing CPR. The correlation between CPR-related attitude and self-efficacy was moderate after the training, suggesting a positive impact on nursing students’ CPR knowledge and confidence [29].

Another noteworthy finding was the significant reduction of anxiety post-simulation training. Given that anxiety can present a substantial barrier to performing CPR effectively, it becomes imperative for nursing students to acquire skills in managing nervousness during stressful situations while also building their confidence [55]. It has been discovered that practicing simulations increases students’ physiological distress [56], a randomized controlled study concluded that HFS in cardiac auscultation was more effective than traditional training in minimizing students’ anxiety levels [26]. Another study examined the effects of high-fidelity clinical simulation during cardiopulmonary resuscitation (CPR) training on stress and anxiety in two groups of nursing students (experienced and inexperienced). Before the first simulated case scenario, all students experienced increased tension and anxiety, but all study groups had lower stress, and anxiety afterward [57]. This suggests that high-fidelity clinical simulation can effectively reduce stress and anxiety in nursing students during CPR training, regardless of their prior experience [57]. The reduction of anxiety levels among students in this study could be attributed to the high self-confidence they gained through the HFS which created an atmosphere similar to the real clinical setting [58].

The limitation of this study comes from the limited possibility of generalizing its findings to all undergraduate nursing students in Palestine as the data were gathered from one nursing college. Another limitation was inherent in the quasi-experimental design as this study did not use a control group for comparison. Additionally, the study relied on a convenient sample and lacked randomization.

## Conclusion

This study revealed the positive effect of using the ACLS Simulation-based training on improving the nursing students’ self-efficacy, and attitude, and decreasing the level of anxiety. The effectiveness of HFS as an educational strategy for future patient safety care is evident as it provides students with a realistic learning environment, ultimately leading to improved patient care outcomes. Therefore, it is highly recommended that HFS be integrated into nursing college labs. Additionally, nursing students rarely have the opportunity to experience resuscitation cases in a clinical setting, making simulation labs a crucial training requirement, and further highlighting the significance of HFS in nursing education. With the integration of HFS in nursing programs, students can gain practical experience and develop the necessary skills to manage emergencies effectively.

## Data Availability

The available datasets, utilized and analyzed for the current study can be obtained from the corresponding author upon making a reasonable request in this regard.

## References

[1] Rano Mal P, Sunnel P. Out-of-hospital cardiac arrest (OHCA): a critical healthcare problem. EC Emerg Med Crit Care. 2019;3(4):197–204.

[2] Mozaffarian D (2015). Heart Disease and Stroke statistics–2015 update: a report from the American Heart Association. Circulation.

[3] Goldberger ZD (2012). Duration of resuscitation efforts and survival after in-hospital Cardiac Arrest: an observational study. Lancet.

[4] Jentzer JC (2016). Improving Survival from Cardiac Arrest: a review of Contemporary Practice and challenges. Ann Emerg Med.

[5] Saramma P et al. *Assessment of long-term impact of formal certified cardiopulmonary resuscitation training program among nurses*. 2016. 20(4):226 10.4103/0972-5229.180043. 10.4103/0972-5229.180043 PMC4906335 27303137

[6] Luciano BA et al. *Necessity of immediate cardiopulmonary resuscitation in trauma emergency* 2010. 5: 1–3. 10.1186/1749-7922-5-25. 10.1186/1749-7922-5-25 PMC2936878 20738879

[7] Hasegawa T (2014). Relationship between weight of rescuer and quality of chest compression during cardiopulmonary resuscitation. J Physiol Anthropol.

[8] Guetterman TC (2019). Nursing roles for in-hospital Cardiac Arrest response: higher versus lower performing hospitals. BMJ Qual Saf.

[9] Tastan S (2017). The effects of music on the cardiac resuscitation education of nursing students. Int Emerg Nurs.

[10] Alconero-Camarero AR (2016). Clinical simulation as a learning tool in undergraduate nursing: validation of a questionnaire. Nurse Educ Today.

[11] Sanagoo A, Araghian F, Mojarad, Jooybari L (2020). Title: clinical simulators: a suitable solution for clinical training of nurses during the corona epidemic. Iran J Med Educ.

[12] Shin S, Park J-H, Kim J-H (2015). Effectiveness of patient simulation in nursing education: meta-analysis. Nurse Educ Today.

[13] Roh YS, Issenberg SB (2014). Association of cardiopulmonary resuscitation psychomotor skills with knowledge and self-efficacy in nursing students. Int J Nurs Pract.

[14] D’Souza MS, Arjunan P, Venkatesaperumal R (2017). High fidelity simulation in nursing education. Int J Health Sci Res.

[15] Cant RP, Cooper SJ (2017). Use of simulation-based learning in undergraduate nurse education: an umbrella systematic review. Nurse Educ Today.

[16] Haddeland K (2018). Nursing students managing deteriorating patients: a systematic review and meta-analysis. Clin Simul Nurs.

[17] La Cerra C (2019). Effects of high-fidelity simulation based on life-threatening clinical condition scenarios on learning outcomes of undergraduate and postgraduate nursing students: a systematic review and meta-analysis. BMJ open.

[18] Ihunanya OM (2020). Knowledge, attitude and practice of cardiopulmonary resuscitation among nurses in Babcock university teaching hospital in Ilishan-Remo, Ogun State, Nigeria. Int J Caring Sci.

[19] Meakim C, et al. Standards of best practice: Simulation standard I: terminology. Clin Simul Nurs. 2013;9(6):S3–S11. 10.1016/j.ecns.2013.04.001.

[20] Fluharty L (2012). A multisite, multi–academic track evaluation of end-of-life simulation for nursing education. Clin Simul Nurs.

[21] Sok SR (2020). Effects of a simulation-based CPR training program on knowledge, performance, and stress in clinical nurses. J Continuing Educ Nurs.

[22] Lendahls L, Oscarsson MG (2017). Midwifery students’ experiences of simulation-and skills training. Nurse Educ Today.

[23] Kaldheim HKA (2019). Use of simulation-based learning among perioperative nurses and students: a scoping review. Nurse Educ Today.

[24] Kim JW (2016). Improvement in trainees’ attitude and resuscitation quality with repeated cardiopulmonary resuscitation training: cross-sectional simulation study. Simul Healthc.

[25] Hendy A et al. Self-assessed capabilities, attitudes, and stress among pediatric nurses in relation to cardiopulmonary resuscitation. J Multidisciplinary Healthc, 2023:603–11. 10.2147/JMDH.S401939 PMC9990508 36896454

[26] Doğru BV, Aydın LZ (2020). The effects of training with simulation on knowledge, skill and anxiety levels of the nursing students in terms of cardiac auscultation: a randomized controlled study. Nurse Educ Today.

[27] Seol J, Lee O (2020). Effects of cardiopulmonary resuscitation training for Mozambican nursing students in a low-resource setting: an intervention study. Nurse Educ Today.

[28] Brennan R, Hajjeh R, Al-Mandhari A (2022). Responding to health emergencies in the Eastern Mediterranean region in times of conflict. The Lancet.

[29] Park G (2015). The Effect of basic cardiopulmonary resuscitation training on cardiopulmonary resuscitation knowledge, attitude, and self-efficacy of nursing students. Adv Sci Technol Lett (Healthcare Nursing).

[30] Carey JM, Rossler K. The how when why of high fidelity simulation. In: StatPearls. StatPearls Publishing; 2020. 32644739

[31] Salik I, Paige JT. Debriefing the interprofessional team in medical simulation 2020. 32119413

[32] Sharma SK (2020). How to calculate sample size for observational and experimental nursing research studies. Natl J Physiol Pharm Pharmacol.

[33] Faul F (2007). G* power 3: a flexible statistical power analysis program for the social, behavioral, and biomedical sciences. Behav Res Methods.

[34] Cook DA (2013). Comparative effectiveness of instructional design features in simulation-based education: systematic review and meta-analysis. Med Teach.

[35] American Heart Association [AHA] (2016). WHAT’s NEW & WHY. Important changes in the 2015 AHA guidelines Update. JEMS.

[36] Roh YS, Lim EJ, Issenberg SB (2016). Effects of an integrated simulation-based resuscitation skills training with clinical practicum on mastery learning and self-efficacy in nursing students. Collegian.

[37] Hernández-Padilla J (2016). Development and psychometric assessment of the basic resuscitation skills self-efficacy scale. Eur J Cardiovasc Nurs.

[38] Cho H. *Analysis of nurses’ attitude toward basic life support and influencing factors* Unpublished master’s thesis, Yonsei University, Seoul, 2008.

[39] Moon H, Hyun HS (2019). Nursing students’ knowledge, attitude, self-efficacy in blended learning of cardiopulmonary resuscitation: a randomized controlled trial. BMC Med Educ.

[40] Youssef N et al. The internal reliability and construct validity of the evidence-based practice questionnaire (EBPQ): Evidence from healthcare professionals in the Eastern Mediterranean Region. Healthcare. MDPI 2023;11(15):2168. 10.3390/healthcare11152168. 10.3390/healthcare11152168 PMC10419240 37570408

[41] Hermawan R, Septian MD, Hayat H (2022). Impacts of implementing the Learning Policy on Vocational Higher Education in Post Covid-19 pandemic. AL-ISHLAH: Jurnal Pendidikan.

[42] Oner N, Le Compte A (1983). State-trait handbook of anxiety inventory.

[43] Spielberger D et al. State-trait anxiety inventory. Palo Alto, CA: Consulting Psychologists Press; 1983. 10.1037/t06496-000.

[44] Spielberger CD et al. State-trait anxiety inventory for adults: Manual, instrument and scoring guide. 2015: Mind Garden. 10.1037/t06496-000.

[45] Azizi M (2022). A comparison of the effects of teaching through simulation and the traditional method on nursing students’ self-efficacy skills and clinical performance: a quasi-experimental study. BMC Nurs.

[46] Olaussen C (2022). Integrating simulation training during clinical practice in nursing homes: an experimental study of nursing students’ knowledge acquisition, self-efficacy and learning needs. BMC Nurs.

[47] Al-Kalaldeh M, Al-Olime Sa (2022). Promoting nurses’ self-efficacy in Advanced Cardiac Life Support through High-Fidelity Simulation. J Continuing Educ Nurs.

[48] Demirtas A (2021). Effectiveness of simulation-based cardiopulmonary resuscitation training programs on fourth-year nursing students. Australasian Emerg Care.

[49] Seo YH, Cho KA. Effect of Korean advanced life support education on non-technical and technical skills of nursing students: a pilot study. Healthcare. MDPI 2021;9(10):1253. 10.3390/healthcare9101253. 10.3390/healthcare9101253 PMC8535704 34682933

[50] Kiernan LCJN. Evaluating competence and confidence using simulation technology. Nursing. 2018;48(10):45. 10.3390/healthcare9101253. 10.1097/01.NURSE.0000545022.36908.f3 PMC6155363 30211760

[51] Dick-Smith F et al. Basic life support training for undergraduate nursing students: An integrative review. Nurse Educ Pract. 2021;50:102957. 10.1016/j.nepr.2020.102957. 10.1016/j.nepr.2020.102957 33421680

[52] Shaban S (2016). Factors influencing medical students′ self-assessment of examination performance accuracy: a United Arab Emirates study. Educ Health.

[53] Ibrahim AF et al. The relationship between undergraduate nursing students’ satisfaction about clinical learning environment and their competency self-efficacy. J Nurs Educ Pract. 2019;9(11):92. 10.5430/jnep.v9n11p92.

[54] Mohamed F, Mahmoud F, Abdel-Salam A (2017). Effect of High-Fidelity Simulation Training on Pediatric nursing student’s knowledge, practice and attitude regarding cardiopulmonary resuscitation. Egypt J Health Care.

[55] Asken MJ (2021). Interns’ anticipatory anxiety about cardiopulmonary resuscitation: reducing it while bolstering confidence with psychological skills training. Intern Emerg Med.

[56] McGuire K, Lorenz R (2018). Effect of simulation on learner stress as measured by cortisol: an integrative review. Nurse Educ.

[57] Fernández-Ayuso D (2018). The modification of vital signs according to nursing students’ experiences undergoing cardiopulmonary resuscitation training via high-fidelity simulation: quasi-experimental study. JMIR Serious Games.

[58] Labrague LJ et al. High-fidelity simulation and nursing students’ anxiety and self‐confidence: A systematic review. Nursing Forum. 2019. Wiley Online Library. 10.1111/nuf.12337. 10.1111/nuf.12337 30852844

